# Comparison of the efficacy of the early LI-SWT plus daily tadalafil with daily tadalafil only as penile rehabilitation for postprostatectomy erectile dysfunction

**DOI:** 10.1038/s41443-022-00560-w

**Published:** 2022-03-28

**Authors:** Se Won Jang, Eun Hye Lee, So Young Chun, Yun-Sok Ha, Seock Hwan Choi, Jun Nyung Lee, Bum Soo Kim, Hyun Tae Kim, See Hyung Kim, Tae-Hwan Kim, Eun Sang Yoo, Jae-Wook Chung, Tae Gyun Kwon

**Affiliations:** 1 Department of Urology, Kyungpook National University Hospital, Daegu, Republic of Korea; 2 Biomedical Research Institute, Kyungpook National University, Daegu, Republic of Korea; 3 Department of Urology, School of Medicine, Kyungpook National University, Daegu, Republic of Korea; 4 Joint Institute for Regenerative Medicine, Kyungpook National University, Daegu, Republic of Korea; 5 Department of Radiology, School of Medicine, Kyungpook National University, Daegu, Republic of Korea

**Keywords:** Erectile dysfunction, Clinical trial design

## Abstract

This study compares the efficacy of the early low-intensity shock wave therapy (LI-SWT) plus daily tadalafil with daily tadalafil only therapy as penile rehabilitation for postprostatectomy erectile dysfunction in patients with prostate cancer who underwent bilateral interfascial nerve-sparing radical prostatectomy (robotic or open). From April 2019 to March 2021, 165 patients were enrolled, and 80 of them successfully completed this prospective study. Daily tadalafil were administered to all the patients. LI-SWT consisted of a total of six sessions. Each session was performed on days 4, 5, 6, and 7, and on the second and fourth weeks after surgery. Each LI-SWT session consisted of 300 shocks at an energy density of 0.09 mJ/mm^2^ and a frequency of 120 shocks per minute that were delivered at each of the five treatment points for 15 min. Thirty-nine patients were treated with tadalafil-only (group A) while 41 were treated with tadalafil and LI-SWT simultaneously (group B). At postoperative 6 months, the proportion of patients with erection hardness scores (EHS) ≥ 3 (4/39 vs. 12/41) was significantly higher in group B (*p* = 0.034), and LI-SWT was the only independent factor for predicting EHS ≥ 3 (OR, 3.621; 95% CI, 1.054–12.437; *p* = 0.041). There were no serious side effects related to early LI-SWT. Early LI-SWT plus daily tadalafil therapy as penile rehabilitation for postprostatectomy erectile dysfunction is thought to be more efficacious than tadalafil only. Further large-scaled randomized controlled trials will be needed to validate these findings.

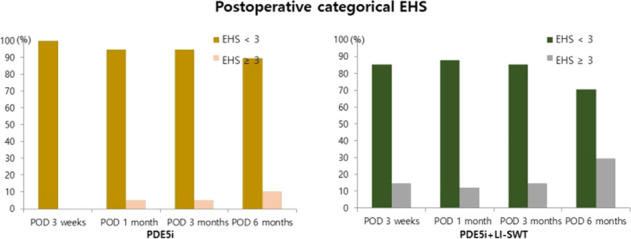

## Introduction

Radical prostatectomy (RP) has evolved with the goal of improving oncologic (cancer-free) and functional (free from urinary incontinence [1] and erectile dysfunction (ED) [2]) outcomes. Although surgical techniques such as nerve-sparing RP have been employed worldwide, postoperative 12-month and 24-month potency rates have been reported to be 54–90% and 63–94%, respectively [3]. In addition, less than 50% of patients returned to baseline erectile function although they were taking phosphodiesterase type-5 inhibitors (PDE5i) [4]. Even after nerve-sparing RP, traumatic injuries to the nerves, known as neurapraxia, occur, which eventually result in the loss of daily and nocturnal erections associated with persistent cavernous hypoxia [5, 6]. These discrepancies associated with the potency rates after RP are attributed to numerous factors, including different baseline characteristics of patients, various nerve-sparing extensions and techniques according to the surgeons’ approach, the definition of potency, and data collection methods [7].

Low-intensity shock wave therapy (LI-SWT) is an emerging new therapeutic modality for ED with promising regenerative effects [8]. A recent meta-analysis of seven randomized controlled trials demonstrated that LI-SWT significantly improved erectile function as revealed by the increased International Index of Erectile Function-5 (IIEF-5) scores [9] and erection hardness scores (EHSs) [10]. LI-SWT can induce microcellular traumatic injuries to the tissues, leading to the release of angiogenic factors and the subsequent neovascularization of the treated tissues [11]. These activities have led to the assumption that if LI-SWT is applied to the corpora cavernosa, it could improve penile blood flow and endothelial function by stimulating angiogenesis in the penis without any adverse effects [12].

Penile rehabilitation is defined as the use of any drug or device at or after RP to maximize erectile function recovery [13]. The goal of penile rehabilitation after RP is to restore preoperative baseline erectile function; however, an optimal penile rehabilitation treatment regimen has not been established. Few studies have evaluated the role of LI-SWT after nerve-sparing RP [14]. This study aims to compare the efficacy and safety of the early LI-SWT plus daily tadalafil to those of daily tadalafil-only therapy as penile rehabilitation for postprostatectomy ED in patients with prostate cancer (PCa) who underwent nerve-sparing RP.

## Materials and methods

### Ethics statement

This prospective study was approved by the Institutional Review Board of Kyungpook National University, School of Medicine, Daegu, Republic of Korea (approval number: KNUH 2018-11-003). The present study was carried out in accordance with the applicable laws and regulations, good clinical practices, and ethical principles outlined in the Declaration of Helsinki. All patients gave their written informed consent after a thorough explanation of the study procedure.

### Study population

**Fig. 1:**
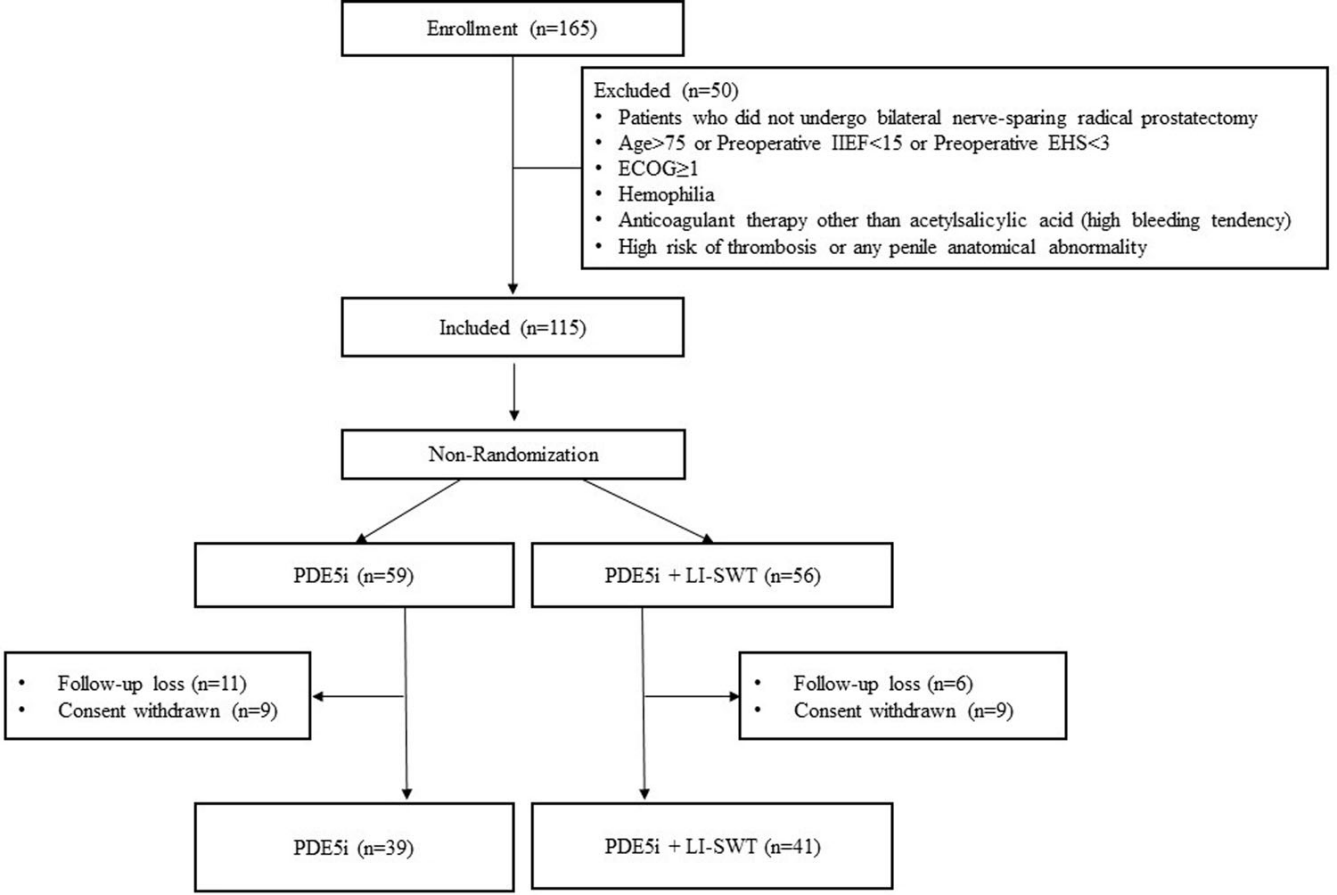
Consort flow chart for study participation. IIEF international index of erectile function, EHS erection hardness score, ECOG Eastern Cooperative Oncology Group performance status, PDE5i phosphodiesterase type-5 inhibitors, LI-SWT low-intensity shock wave therapy.

From April 2019 to March 2021, 165 patients were enrolled in the study. The investigators thoroughly explained the aim of this study to the patients, and only the patients who agreed to the LI-SWT were assigned to one of the two treatment groups. The inclusion criteria are as follows: (1) patients who underwent bilateral interfascial nerve-sparing RP (robotic or open) [15]. (2) patients whose erectile function was expected to recover after RP (age ≤ 75, preoperative IIEF-5 ≥ 15 [16, 17], EHS ≥ 3 [18]). Fifty patients did not meet the inclusion criteria, 17 patients were lost to follow-up, and 18 withdrew their consent; thus, 80 patients completed this prospective study (Fig. 1). The Eastern Cooperative Oncology Group performance status [19] of all patients was 0. We excluded patients with hemophilia, patients who were on anticoagulant therapy other than acetylsalicylic acid (high bleeding tendency), and patients with a high risk of thrombosis or any penile anatomical abnormality. Daily PDE5i (5-mg tadalafil) was given to all patients every day from 1 week to 6 months after RP. Finally, 39 patients were treated with PDE5i only (group A), while 41 patients were treated with PDE5i and LI-SWT simultaneously (group B). The primary endpoint of the study was to confirm the restoration of erectile function (EHS ≥ 3) at 6 months after RP.

### Study protocol

The IIEF-5 questionnaire and EHSs were assessed before surgery and at 3 weeks, 1 month, 3 months, and 6 months postoperatively. All the questionnaires were recorded by research nurses. The investigators were not blinded to the treatment arm. The patients underwent a total of six sessions of LI-SWT. Each session was performed on days 4, 5, 6, and 7, and on the second and fourth weeks RP. Patients who underwent robot-assisted laparoscopic RP had their urethral catheters removed on the 6th day after surgery and they were discharged the same day. This is done on the 7th day after surgery for patients who underwent retropubic RP. Two urologists performed the LI-SWT procedure. LI-SWT was performed by SWJ for inpatients and by JWC for outpatients. During their hospital stay, the patients underwent LI-SWT with their urethral catheters inserted, without any related adverse events.

LI-SWT was performed using ED1000^®^ (MEDISPEC, USA). Each LI-SWT session consisted of 300 shocks at an energy density of 0.09 mJ/mm^2^ and a frequency of 120 shocks per minute that were delivered at each of the five treatment points (distal, mid, and proximal penile shaft, and left and right crura). Each treatment session lasted for 15 min. During each treatment session, patients were asked if they experienced any side effects.

### Statistical analysis

Continuous variables were not normally distributed and compared using the Mann–Whitney test. Comparisons between categorical variables were performed using the Chi-square test or Fisher’s exact test. In addition, univariate and multivariate logistic regression models were used to identify factors that were predictive of the recovery of erectile function after nerve-sparing RP. Logistic regression models were used to generate odds ratios (ORs) with 95% confidence intervals (CIs). Statistical analyses were performed using the Statistical Package for the Social Sciences version 16.0 for Windows (SPSS Inc., Chicago, IL, USA), and *p* < 0.05 was considered statistically significant.

## Results

Table 1 shows the preoperative characteristics of the patients. The median (IQR) age of the study participants was 63.00 (9.00) years. Eighteen (22.5%) patients had diabetes mellitus. The median (IQR) preoperative prostate-specific antigen titer was 6.67 (5.39) ng/ml. Robot-assisted laparoscopic RP was performed on 70 patients (87.5%). The median (IQR) preoperative IIEF-5 score was 18.50 (5.00), and the preoperative EHS was 3.00 (1.00). There were no significant differences in preoperative categorical EHS.

**Table 1:** Preoperative characteristics of the patients.

	Total (*n* = 80) 100%	PDE5i (*n* = 39) 48.8%	PDE5i + LI-SWT (*n* = 41) 51.3%	*p*-value
Age	63.00 (9.00)	66.00 (10.00)	62.00 (8.50)	0.159
Body mass index	24.88 (4.57)	24.36 (5.09)	24.88 (4.45)	0.806
Diabetes mellitus	18 (22.5%)	12 (30.8%)	6 (14.6%)	0.084
Preoperative PSA	6.67 (5.39)	6.23 (4.49)	7.00 (6.55)	0.321
Operative method				0.738^a^
Open	10 (12.5%)	4 (10.3%)	6 (14.6%)	
Robotic	70 (87.5%)	35 (89.7%)	35 (85.4%)	
Preoperative IIEF-5	18.50 (5.00)	18.00 (5.00)	19.00 (4.50)	0.858
Preoperative EHS	3.00 (1.00)	3.00 (1.00)	3.00 (1.00)	0.137
Preoperative EHS (categorical)				0.135
3	53 (66.3%)	29 (74.4%)	24 (58.5%)	
4	27 (33.8%)	10 (25.6%)	17 (41.5%)	

Continuous variables are presented as median value (IQR).

*P*-value according to Mann–Whitney test for continuous variables and Chi-square test for categorical variables, as indicated.

*PSA* prostate-specific antigen, *IIEF* international index of erectile function, *EHS* erection hardness score, *PDE5i* phosphodiesterase type-5 inhibitors, *LI-SWT* low-intensity shock wave therapy.

^a^Fisher’s exact test.

Table 2 details the postoperative pathologic outcomes. Forty-nine patients (61.3%) demonstrated organ-confined (pT2) disease. Only two patients (2.5%) demonstrated pN1. The surgical margin was positive in 31 patients (38.8%). The Gleason score did not differ significantly between the two groups.

**Table 2:** Postoperative pathologic outcomes.

	Total (*n* = 80)	PDE5i (*n* = 39)	PDE5i + LI-SWT (*n* = 41)	*p*-value
pT stage				0.684
Organ confined (pT2)	49 (61.3%)	23 (59.0%)	26 (63.4%)	
Nonorgan confined (≥pT3)	31 (38.8%)	16 (41.0%)	15 (36.6%)	
pN stage				0.494^a^
N0	78 (97.5%)	39 (100.0%)	39 (95.1%)	
N1	2 (2.5%)	0 (0.0%)	2 (4.9%)	
Surgical margin state				0.153
Negative	49 (61.3%)	27 (69.2%)	22 (53.7%)	
Positive	31 (38.8%)	12 (30.8%)	19 (46.3%)	
Gleason score				0.945
6	4 (5.0%)	2 (5.1%)	2 (4.9%)	
7	62 (77.5%)	31 (79.5%)	31 (75.6%)	
8	11 (13.8%)	5 (12.8%)	6 (14.6%)	
9	3 (3.8%)	1 (2.6%)	2 (4.9%)	

*P*-value according to Chi-square test for categorical variables, as indicated.

*PDE5i* phosphodiesterase type-5 inhibitors, *LI-SWT* low-intensity shock wave therapy.

^a^Fisher’s exact test.

**Fig. 2:**
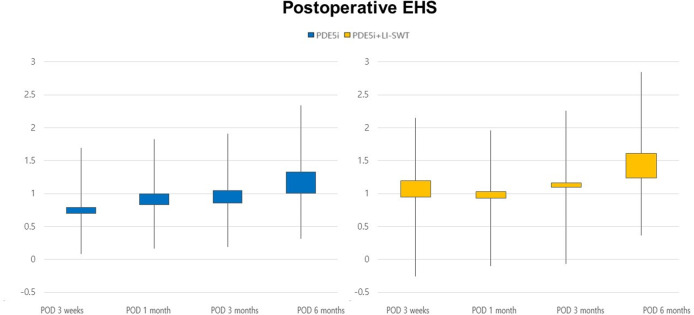
Postoperative erection hardness score. EHS erection hardness score, PDE5i phosphodiesterase type-5 inhibitors, LI-SWT low-intensity shock wave therapy.

EHSs increased gradually at postoperative 3 weeks and 1, 3, and 6 months in both groups (Fig. 2). However, postoperative EHSs were not significantly different between the two groups. The proportion of EHSs (≥ 3) at postoperative 6 months (10.3% vs. 29.3%, *p* = 0.034) was significantly higher in group B (Table 3).

**Table 3:** Postoperative categorical erection hardness score.

	PDE5i (*n* = 39)		PDE5i + LI-SWT (*n* = 41)		*p*-value
EHS, categorical	EHS < 3	EHS ≥ 3	EHS < 3	EHS ≥ 3	
POD 3 weeks	39 (100.0%)	0 (0.0%)	35 (85.4%)	6 (14.6%)	0.026^a^
POD 1 month	37 (94.9%)	2 (5.1%)	36 (87.8%)	5 (12.2%)	0.433^a^
POD 3 months	37 (94.9%)	2 (5.1%)	35 (85.4%)	6 (14.6%)	0.265^a^
POD 6 months	35 (89.7%)	4 (10.3%)	29 (70.7%)	12 (29.3%)	0.034

*P*-value according to Chi-square test for categorical variables, as indicated.

*EHS* erection hardness score, *POD* postoperative day, *PDE5i* phosphodiesterase type-5 inhibitors, *LI-SWT* low-intensity shock wave therapy.

^a^Fisher’s exact test.

Table 4 shows the univariate and multivariate logistic regression model for predicting an EHS ≥ 3 at postoperative 6 months. The application of LI-SWT was the only independent factor for predicting the EHS ≥ 3 at postoperative 6 months (OR, 3.621; 95% CI, 1.054–12.437; *p* = 0.041).

**Table 4:** Univariate and multivariate logistic regression models for predicting the erection hardness score ≥ 3 at 6 months post-operation.

	EHS < 3 (*n* = 64)	EHS ≥ 3 (*n* = 16)	*p* value		OR (95% CI)
			Univariate	Multivariate	
Age	64.50 (8.00)	61.00 (11.25)	0.237	–	–
Body mass index	24.88 (4.96)	24.06 (4.14)	0.799	–	–
Diabetes mellitus	16 (25.0%)	2 (12.5%)	0.503^a^	–	–
OP method			0.677^a^		
Open	9 (14.1%)	1 (6.3%)			
Robot	55 (85.9%)	15 (93.8%)		–	–
Preoperative EHS (categorical)			0.813		
3	42 (65.6%)	11 (68.8%)			
4	22 (34.4%)	5 (31.3%)		–	–
Group A vs. B	29 (45.3%)	12 (75.0%)	0.034	0.041	3.621 (1.054–12.437)

Continuous variables are presented as median value (IQR).

*P*-value according to Mann–Whitney test for continuous variables and Chi-square test for categorical variables, as indicated.

*EHS* erection hardness score, *OP* operation, *LI-SWT* low-intensity shock wave therapy, *OR* odds ratio, *CI* confidence interval.

^a^Fisher’s exact test.

There were no side effects associated with LI-SWT. Three patients complained of adverse events associated with PDE5i. Two patients had hot flushes and one had palpitations. However, there were no serious side effects requiring the discontinuation of PDE5i.

## Discussion

To our best knowledge, this is the first study on penile rehabilitation for postprostatectomy ED following nerve-sparing RP with an “early” application of LI-SWT. We were able to identify a higher EHS in group B during the total study period, and a gradual improvement in erectile function was observed in both treatment groups. Six months after RP (5 months after the end of the final LI-SWT), the proportion of participants with EHS scores of ≥3 was significantly higher in the LI-SWT combined with PDE5i treatment group than in the PDE5i only group. The LI-SWT was found to be safe and did not cause any discomfort.

As described in the introduction section, ED is a common side-effect of RP, including nerve-sparing RP [20]. Older surgical techniques for RP damaged neurovascular bundles completely and permanently, whereas neurapraxia is the common cause of recent postprostatectomy ED after bilateral nerve-sparing RP as a result of surgical manipulations such as coagulation, traction, and compression [7]. Temporary cavernous nerve injuries induce nervous Wallerian degeneration, which results in the denervation of the corpus cavernosum and causes a consequential loss of nocturnal erection [21]. Subsequently, long-term penile hypoxia causes penile structural remodeling with smooth muscle apoptosis, fibrosis, and veno-occlusive dysfunction [22]. Although the recovery of potency after RP is influenced by various factors related to the individual characteristics of the patient, a progressive gradual increase in potency rates has been demonstrated by follow-up evaluations after RP [3].

The most common clinical protocol for penile rehabilitation after RP is regular dosage with PDE5i [23]. Several clinical studies have demonstrated a potential role for PDE5i in the recovery of erectile function, provided it is implemented early after RP [24]. According to Bannowsky et al. [25], at 52 weeks after nerve-sparing RP, 47% of men taking 25-mg sildenafil maintained erectile function sufficient for intercourse compared with 28% of men in the control group (*p* < 0.001). However, other studies have arrived at the opposite conclusion. Pavlovich et al. [26] randomly assigned 100 men who underwent nerve-sparing RP into the nightly sildenafil group and the on-demand placebo group. No significant differences were found in erectile function between treatments at any time point after RP. Other regimens for penile rehabilitation after RP were intracavernous injections and a vacuum erection device [27]. Although these therapies show somewhat favorable results, they were not used routinely for erectile rehabilitation because of the introduction of PDE5i. However, penile rehabilitation attempts for restoring spontaneous erections through scheduled postoperative treatments with erectogenic aids have generally been disappointing [28].

Penile extracorporeal LI-SWT recently emerged as a novel, promising treatment modality for erectile dysfunction [29]. Unlike other currently used treatment modalities for ED, all of which are palliative in nature, LI-SWT aims to restore the erectile function mechanism by enabling natural or spontaneous penile tumescence [30]. Although the potential mechanism of action of LI-SWT in the treatment of ED is not clearly understood, it is hypothesized on the basis of the current literature that the shockwaves trigger cellular pathways, increasing the expression of growth factors and endothelial nitric oxide synthase and resulting in angiogenesis and the regeneration of nerve fibers [31–34]. Although more randomized controlled trials are warranted to overcome study limitations and conflicts between the results of existing studies before the widespread acceptance of LI-SWT as the standard of care for ED [30, 35–38], it is generally accepted that shock waves interact with targeted tissues and induce a cascade of biological reactions that involve the release of various growth factors and the subsequent neovascularization of penile tissue [39].

In a series of clinical trials, including randomized double-blind sham-controlled studies, LI-SWT has been shown to have a substantial effect on penile hemodynamics and erectile function in patients with vasculogenic ED without any significant adverse effects [35, 40]. Vasculogenic ED is the main study object of clinical LI-SWT; however, studies on post-RP ED are rarely referred to in the current literature [21].

In a pilot study carried out by Frey et al. [14] that included 16 patients who had more than a 1-year history of bilateral nerve-sparing RP, patients with ED received two LI-SWT sessions every other week for 6 weeks. Each session included 1000 shock waves with energy densities of 20, 15, and 12 mJ/mm^2^, which was applied to the root of the penis, the shaft, and at a few millimeters proximal to the glans, respectively, for a total of 3,000 shock waves and a frequency of 5 Hz. This study concluded that LI-SWT may enhance erectile function, with median improvement in five-item IIEF scores of 3.5 (range: −1 to 8; *p* = 0.0049) and 1 (range: −3 to 14; *p* = 0.046) at 1 month and 1 year after treatment, respectively. The use of erectogenic aids was not prohibited in this study. The combination of LI-SWT and medicated urethral systems for erections and PDE5i appeared to be somewhat beneficial for the recovery of erectile function. To our knowledge, it is the only published study to focus specifically on LI-SWT for post-RP ED.

Interestingly, a similar study by Zewin et al. [23] evaluated the role of LI-SWT in penile rehabilitation after nerve-sparing radical cystoprostatectomy. This study included 128 sexually active men with muscle-invasive bladder cancer and categorized them into three groups: the LI-SWT group (42 patients), PDE5i group (43 patients), and control group (43 patients). Potency recovery rates at 9 months were 76.2%, 79.1%, and 60.5% in LI-SWT, PDE5i, and control groups, respectively. There was a statistically significant increase in IIEF scores and EHSs for all study groups during all follow-up periods (*p* < 0.001). However, there was no significant difference between the three groups during all follow-up periods. Although the difference was not statistically significant, the study was of clinical importance. LI-SWT is safe as an oral PDE5i in penile rehabilitation post nerve-sparing radical cystoprostatectomy. However, the exact time when LI-SWT should be started was not stated clearly. Unlike the two studies described above, this study focused on the early introduction of LI-SWT after RP. In a previous study, there was a significantly long period after RP until the initiation of LI-SWT, which is why we hypothesized that LI-SWT at an earlier stage after RP could prevent penile fibrosis caused by long-term hypoxia due to the loss of erections.

To the best of our knowledge, this is the first prospective study to evaluate the efficacy and safety of the early application of LI-SWT for ED in patients with PCa who underwent nerve-sparing RP and compared it with the efficacy and safety of oral PDE5i. However, this study has several limitations. First, the absence of a control group (not using PDE5i in combination with LI-SWT) and an only-LI-SWT treatment group or sham treatment group constitutes a significant limitation. It was also difficult to restrict the use of PDE5i during the study period. Because most of the patients wanted to regain their baseline erectile function, restricting PDE5i use would not be ethically justifiable since it would have a potential negative impact on patients’ sexual activities during the study. The small patient cohort and the non-randomization of the treatment groups may have caused selection bias. Missing follow-up results for more than one year was another drawback. As the IIEF-5 scores of many patients were missing, only EHS was analyzed during the follow-up periods. The total number of PDE5i pills used was not uniform between patients, and it was difficult to expect patients’ exact compliance in taking PDE5i. Therefore, any interpretation of the present study should be done with caution. Furthermore, studies that entail objective assessments such as dynamic duplex ultrasound of the penis or nocturnal penile tumescence and rigidity tests will be necessary to confirm the validity of this study. A large-scale study is warranted to confirm our results and to determine the value of LI-SWT as a treatment modality for ED after RP.

Nevertheless, early LI-SWT plus daily tadalafil therapy as penile rehabilitation for postprostatectomy ED is thought to be more efficacious than tadalafil only. There were no serious side effects related to early LI-SWT. Although these improvements were not applied to unassisted full and hard erections sufficient for intercourse in most patients, the present study is of clinical importance as this is the first trial to demonstrate the efficacy of the early application of LI-SWT. To overcome non-randomized, non-controlled nature of these study, further large-scaled randomized controlled trials will be needed to validate these findings.

## Data Availability

All relevant data are within the manuscript and its Supporting Information files.
