# Correction: Comparison of the efficacy of the early LI-SWT plus daily tadalafil with daily tadalafil only as penile rehabilitation for postprostatectomy erectile dysfunction

**DOI:** 10.1038/s41443-022-00595-z

**Published:** 2022-07-12

**Authors:** Se Won Jang, Eun Hye Lee, So Young Chun, Yun-Sok Ha, Seock Hwan Choi, Jun Nyung Lee, Bum Soo Kim, Hyun Tae Kim, See Hyung Kim, Tae-Hwan Kim, Eun Sang Yoo, Jae-Wook Chung, Tae Gyun Kwon

**Affiliations:** 1 Department of Urology, Kyungpook National University Hospital, Daegu, Republic of Korea; 2 Biomedical Research Institute, Kyungpook National University, Daegu, Republic of Korea; 3 Department of Urology, School of Medicine, Kyungpook National University, Daegu, Republic of Korea; 4 Joint Institute for Regenerative Medicine, Kyungpook National University, Daegu, Republic of Korea; 5 Department of Radiology, School of Medicine, Kyungpook National University, Daegu, Republic of Korea

**Keywords:** Erectile dysfunction, Clinical trial design

Correction to: *International Journal of Impotence* Research

10.1038/s41443-022-00560-w, published online 28 March 2022

In this article the heading for table 4 should have read “Univariate and multivariate logistic regression models for predicting the erection hardness score ≥ 3 at 6 months post-operation.” The original article has been corrected.

